# Magnesium sulfate enhances the effect of the peripheral analgesic cocktail in total knee arthroplasty: a systematic review and meta-analysis of randomized controlled trials

**DOI:** 10.1530/EOR-23-0185

**Published:** 2024-09-02

**Authors:** Qiuyuan Wang, Feng Li, Yidan Yang, Chen Yue, Jiayi Guo

**Affiliations:** 1 Department of Evidence-based Medicine, Luoyang Orthopedic Hospital of Henan Province. Orthopedic Hospital of Henan Province, Luoyang, China; 2 Department of Bone And Joint Diseases, Luoyang Orthopedic Hospital of Henan Province. Orthopedic Hospital of Henan Province, Luoyang, China

**Keywords:** magnesium sulfate, meta-analysis, peripheral analgesic cocktail, total knee arthroplasty

## Abstract

**Purpose:**

- Although magnesium sulfate (MgSO_4_) is widely used as an analgesic adjuvant to peripheral analgesic cocktails, its efficacy in total knee arthroplasty (TKA) is still controversial. Therefore, we systematically reviewed and meta-analyzed the literature to assess the analgesic efficacy of MgSO_4_ as an adjuvant to the analgesic cocktail in TKA.

**Methods:**

- The PubMed, EMBASE, Web of Science, and Cochrane Library databases were searched. The meta-analysis was performed according to the PRISMA guidelines. Data were qualitatively synthesized or meta-analyzed using a random-effects model.

**Results:**

- Five randomized controlled trials involving 432 patients were included. Meta-analyses detected significant differences between the MgSO_4_ and control groups in the visual analog scale (VAS) pain scores (rest) at 6, 12, and 24 h postoperatively; VAS pain scores (motion) at 12, 24, and 48 h postoperatively; morphine consumption within 24 h, 24–48 h, and during the total hospitalization period; time to first rescue analgesia after TKA; and length of hospital stay. Regarding the functional recovery, the meta-analysis demonstrated significant differences between groups in terms of knee range of motion on postoperative day 1; daily mobilization distance on postoperative day 1; and daily mobilization distance. There was no significant intergroup difference in surgical complications.

**Conclusion:**

- The findings suggest that MgSO_4_ is a promising adjunct to the analgesic cocktail, achieving significant improvements in pain scores and total opioid consumption during the early postoperative period after TKA.

## Introduction

Total knee arthroplasty (TKA) is an effective surgical intervention aimed at enhancing function and alleviating pain in patients with end-stage knee joint disorders. In the USA, 480 958 TKA procedures were conducted during 2019, and projections suggest a sustained increase in the volume of these procedures in the coming years (1). More than 60% of patients undergoing TKA experience pronounced postoperative pain (2). Pain following TKA is a predominant factor contributing to the extension of the length of hospital stay (LOS) and functional recovery, and frequently serves as a primary cause for patient readmission (3). Therefore, effective postoperative pain management is crucial for early recovery and enhanced patient satisfaction. As a pivotal element within the realm of multimodal analgesia, peripheral analgesia techniques have been extensively used in TKA throughout the past decade (2). These techniques encompass periarticular local anesthetic injection and a range of peripheral nerve blocks.

The standard pharmaceutical approach for peripheral analgesia typically involves combinations of local anesthetics and other adjuvants such as epinephrine and glucocorticoids, which enhance analgesic efficacy (4). The prepared mixed formulations are commonly referred to as ‘cocktails’ by researchers. However, there remains no gold standard for the precise composition and dosing of pharmaceutical components for optimal analgesic cocktails, resulting in divergent efficacy outcomes reported among different medical institutions (5, 6). Consequently, researchers are diligently investigating a more refined composition for analgesic cocktails.

Despite the addition of various adjuvants, including epinephrine, clonidine, morphine, non-steroidal anti-inflammatory drugs, and corticosteroids, to local anesthetics to enhance their analgesic effects (7), the prolongation of postoperative analgesia remains constrained. Magnesium functions as an antagonist of N-methyl-d-aspartate (NMDA) receptors (8) and is an effective analgesic adjuvant for postoperative pain management (9). Magnesium sulfate (MgSO_4_) has undergone thorough investigation in various orthopedic procedures, and recent studies have evaluated the use of MgSO_4_ as an additive to the drug cocktail in TKA (10, 11, 12, 13, 14). However, the effectiveness of MgSO_4_ as an additive to the drug cocktail in TKA is unclear. Studies have reported that incorporating MgSO_4_ into the peripheral analgesic cocktail after TKA prolongs postoperative analgesia, decreases opioid consumption, and alleviates initial postoperative pain (10, 11, 12, 13), and has the potential to enhance knee joint function (12, 13). However, one study reported that the supplementation of MgSO_4_ to the peripheral analgesic cocktail had no significant analgesic advantage in patients undergoing TKA (14).

There is a consensus that medical interventions should adhere to an evidence-based approach, which entails drawing conclusions from meticulously conducted randomized controlled trials (RCTs) and meta-analyses that meet the highest contemporary scientific standards. Thus, we performed a systematic review and meta-analysis of RCTs to evaluate the impact of MgSO_4_ as an additive drug to the peripheral analgesic cocktail in TKA.

## Methods

A systematic search was conducted to identify RCTs following the methodologies outlined in the *Cochrane Handbook for Systematic Reviews of Interventions* (15). This meta-analysis was performed in accordance with the PRISMA statement (16). The review was registered in the PROSPERO, with the registration number CRD42023470356. As all analyses were conducted using data from previously published studies, ethical approval was not required.

### Search strategy

We conducted a systematic literature search of major electronic databases, namely PubMed, EMBASE, Web of Science, and the Cochrane Library. The last search date was August 15, 2023. There were no language or publication year restrictions. The search strategy used a combination of medical subject headings and keywords related to ‘Magnesium Sulfate’ and ‘Arthroplasty, Replacement, Knee’. The search terms were ‘Magnesium Sulfate’, ‘Magnesium’, ‘Magnesium Sulfate, Heptahydrate’, ‘Arthroplasty, Replacement, Knee’, ‘Arthroplasty, Knee Replacement’, ‘Knee Replacement Arthroplasty’, ‘Knee Arthroplasty, Total’, ‘Arthroplasty, Total Knee’, ‘Total Knee Arthroplasty’, ‘Replacement, Total Knee’, and ‘Total Knee Replacement’ (Supplemental Table 1, see section on supplementary materials given at the end of this article). Search terms were combined using the Boolean operators ‘AND’ or ‘OR’. The reference lists of relevant articles were meticulously searched to identify additional trials.

### Inclusion criteria and study selection

A study was considered eligible for inclusion if: i) it was an RCT comparing TKA patients who received a topical MgSO_4_ in combination with a peripheral analgesic cocktail to TKA patients who received a peripheral analgesic cocktail alone; and ii) it reported at least one of the following outcomes: pain score, opioid consumption, functional evaluation of the knee joint, length of hospital stay (LOS), or adverse events. A study was excluded if it: i) was based on the use of an intravenous analgesic infusion with added magnesium; ii) compared the effect of MgSO_4_ with a blank/placebo; iii) involved incomplete data, or the full-text article was not available, or was an animal study; or iv) was published as a review, letter, or conference abstract.

All retrieved studies were imported into EndNote 9 (Thomson Scientific, Stamford, CT, USA). The exclusion of irrelevant studies based on titles and abstracts was independently conducted by the same two authors who had conducted the initial database searches. Subsequently, the full texts of studies that met the predefined inclusion criteria underwent thorough screening, and a final determination on study eligibility was reached. Any discrepancies between the two authors were resolved through discussion with a third author.

### Data extraction

The same two authors independently extracted data from selected studies, including the first author(s)’ name, country, publication year, numbers of patients in the intervention and control groups, sample size, dosage of MgSO_4_ added in the trial group, and composition of the peripheral anesthetic cocktail administered in the control group. Indicators of pain consisted of the visual analog scale (VAS) score at rest or during motion within 72 h after surgery, postoperative morphine consumption for rescue analgesia, and time to first rescue analgesia. Any opioid consumption was converted to the oral morphine equivalent (17). Knee functional recovery was assessed by knee range of motion, daily mobilization distance, and time to first straight leg raising. Additionally, we assessed the LOS and major surgical complications (postoperative nausea or vomiting (PONV), wound complications, deep vein thrombosis, chronic pain, pruritus, and sedation).

Data were independently extracted and inputted into an Excel spreadsheet, and the risk of bias for each eligible article was assessed by two authors. Disagreements were resolved during meetings with all authors.

### Quality assessment

The Cochrane risk-of-bias tool was used to assess the quality and risk of bias of the included RCTs (15). We systematically evaluated various aspects of methodological rigor, namely random sequence generation, allocation concealment, blinding of participants and personnel, blind outcome assessment, handling of incomplete outcome data, selective reporting, and potential sources of bias. The overall quality of the included studies was categorized as a low, unclear, or high risk of bias. The quality assessment was conducted independently by two authors, and any discrepancies were resolved through discussions.

The quality of evidence for the outcomes in the current meta-analysis was assessed using the Recommendations Assessment, Development and Evaluation system, which considers the following elements: risk of bias, inconsistency, indirectness, imprecision, and publication bias (18). The level of evidence was categorized as high, moderate, low, and very low.

### Statistical analysis

The statistical analysis was performed using RevMan software, version 5.4, provided by the Cochrane Collaboration. All outcomes were meta-analyzed using a random-effects model. Dichotomous data were analyzed using risk ratios (RRs). Continuous data measured on congruent scales were presented as mean differences (MDs) with corresponding 95% CIs, while standardized mean differences (SMDs) were utilized for continuous outcomes assessed on different scales with 95% CIs. Results that were originally presented as the median and interquartile range were transformed into the mean and standard deviation following the guidelines outlined in The Cochrane Handbook (15) when applicable. Statistical significance was set at *P* < 0.05. Heterogeneity across studies was assessed using the *I*^2^ test, with *I*^2^ > 50% defined as substantial heterogeneity.

## Result

### Search results and characteristics of included studies

**Figure 1:**
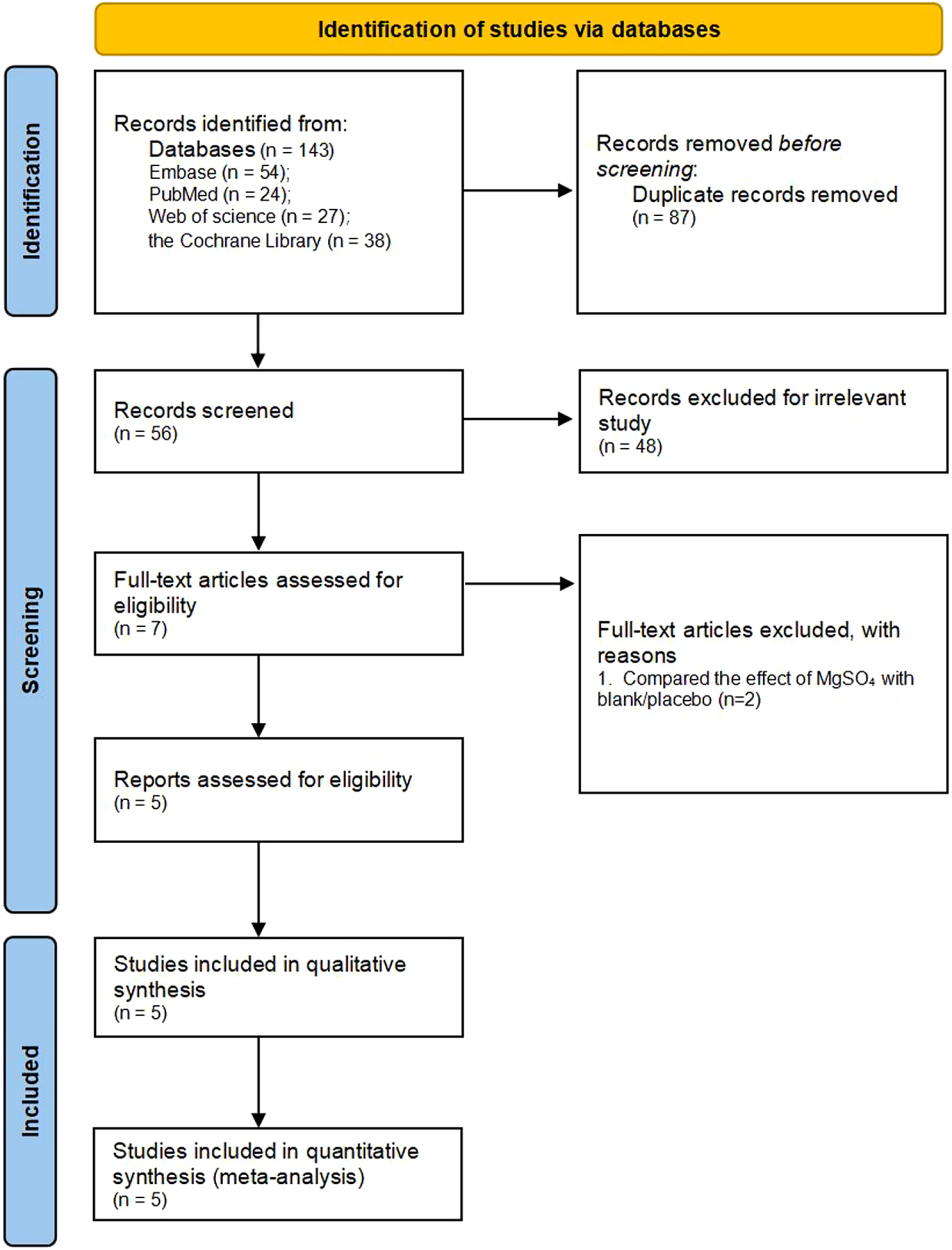
Search result and study selection procedure.

The systematic searches of the PubMed, Embase, Web of Science, and Cochrane Library databases yielded 24, 54, 27, and 38 citations, respectively. After removing duplicates, 56 articles were subjected to initial screening, wherein the titles and abstracts were independently assessed by two authors. Subsequently, the full text of seven RCTs was read. Two of these RCTs were excluded because they compared the effect of MgSO_4_ with a blank/placebo (19, 20). Consequently, five RCTs involving 432 patients were included in the meta-analysis. The details of the identification, inclusion, and exclusion of studies are illustrated in Fig. 1.

All included RCTs had been published since 2021. Three RCTs were performed in China, one was performed in the USA, and one was performed in Canada. The baseline characteristics of the included RCTs are summarized in Table 1.

**Table 1:** Description of studies and participants.

Reference	Country	Year	Number of patients			Dosage of MgSO_4_ in IG	Composition of peripheral anesthetic solution cocktail in CG
			IG	CG	Total		
Zhao *et al.* (11)	China	2021	30	30	60	250 mg	20 mL levobupivacaine 50 mg + triamcinolone 25 mg + 0.9% normal saline
Zoratto *et al.* (15)	Canada	2021	41	39	80	2 g of 10% MgSO_4_	10 mL ropivacaine 0.5% + 10 mL normal saline
Choi *et al.* (12)	America	2022	49	53	102	150 mg (0.3 mL)	30 mL 0.25% bupivacaine and 0.3 mL sterile saline
Wang *et al.* (13)	China	2023	50	50	100	2.5 mg/mL	100 mL ropivacaine (0.2%), epinephrine (2.0 mg/mL), and dexamethasone (0.1 mg/mL)
Zhao *et al.* (14)	China	2023	45	45	90	2.5 mg/mL	100 mL 0.2% ropivacaine, dexamethasone (0.1 mg/mL), and epinephrine (2.0 μg/mL)

CG, control group; IG, intervention group; MgSO4, magnesium sulfate.

**Figure 2:**
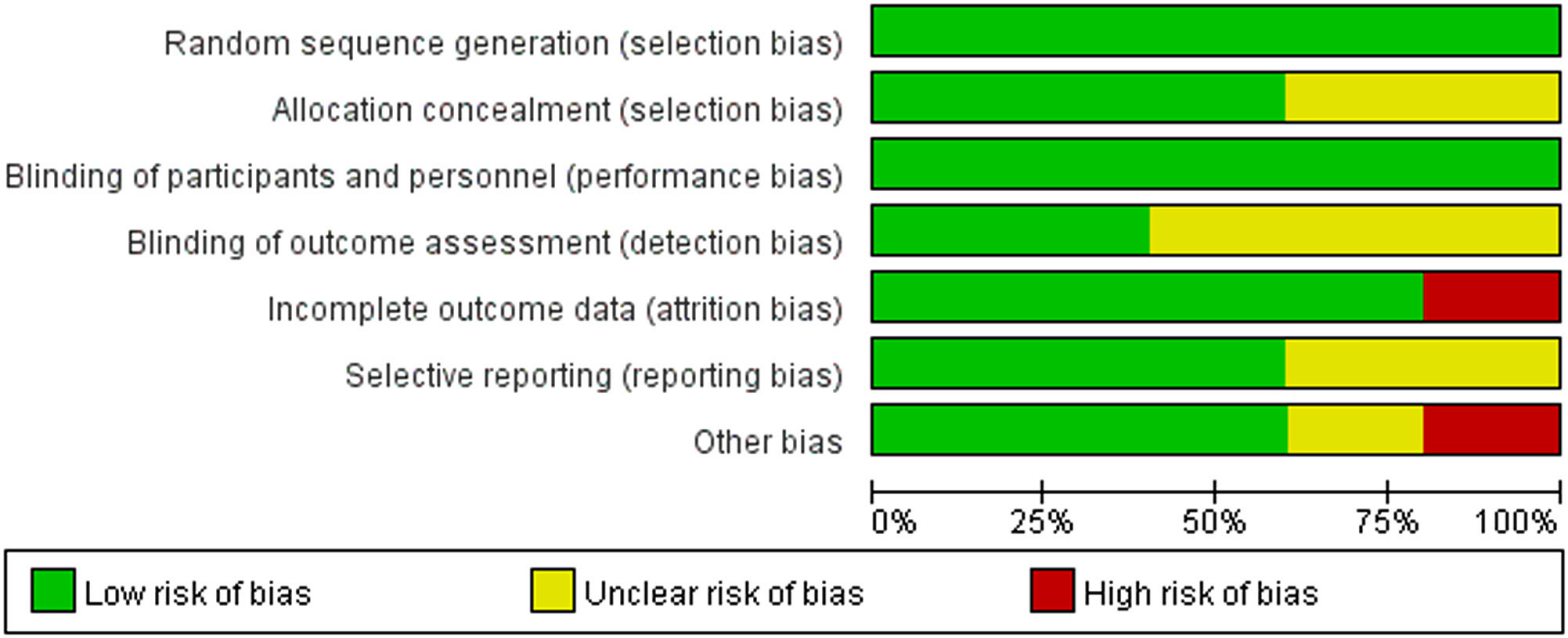
Risk of bias in included studies. Green indicates a low risk, yellow indicates an unclear risk, and red indicates a high risk of bias.

All RCTs (10, 11, 12, 13, 14) adhered to clear inclusion and exclusion criteria and thoroughly reported the randomization methodology, with a description of the use of computer-generated randomization. In three RCTs (12, 13, 14), allocation concealment was achieved using sealed envelopes. All RCTs used double-blinding, and two (12, 13) made earnest attempts to blind the assessors. One RCT (14) had a high risk of bias in terms of incomplete outcome data. Two studies (12, 13) had an unclear risk of bias regarding selective reporting. In addition, one RCT (11) had an unclear risk of bias, and one (14) had a high risk of bias. The methodological quality assessment is summarized in Fig. 2. The quality of evidence, as assessed by the GRADE, is presented in Supplementary Table 2.

### Pain scores

**Figure 3:**
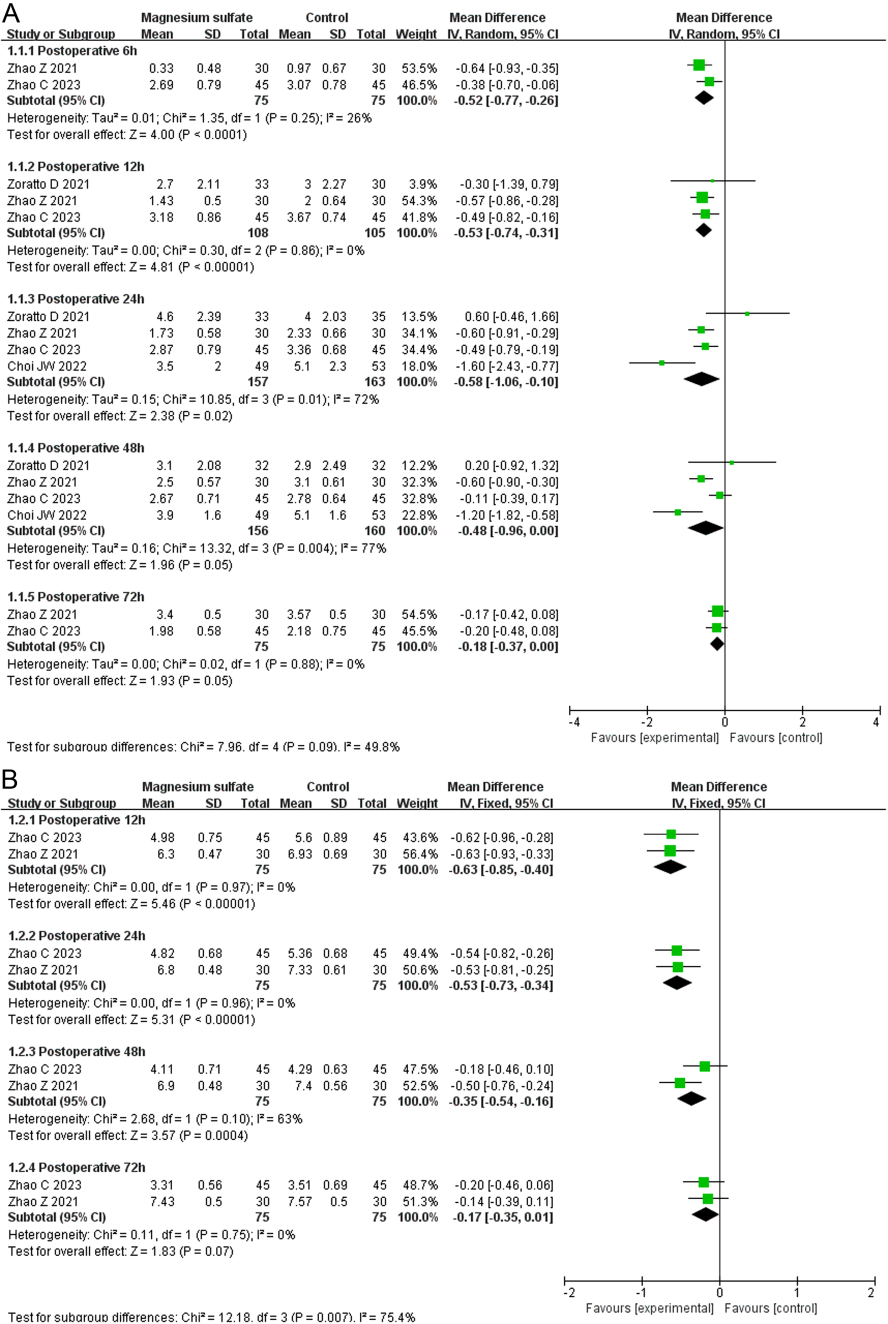
Forest plots displaying the mean differences in 6- to 72-h postoperative visual analog scale scores between the magnesium sulfate and control groups A) at rest and B) during motion. Green squares with horizontal lines represent the mean differences and 95% CIs for each trial. Black tiles serve as graphical representations of the mean differences at distinct time intervals (11, 12, 14, 15).

VAS scores (at rest) were investigated in four RCTs involving 320 patients (10, 11, 13, 14). As shown in Fig. 3A, the meta-analysis demonstrated that the VAS scores (at rest) were significantly lower in the MgSO_4_ group than the control group at 6 h postoperatively (MD: −0.52, 95% CI: −0.77 to −0.26, *I*^2^ = 26%, GRADE: MODERATE), 12 h postoperatively (MD: −0.53, 95% CI: −0.74 to −0.31, *I*^2^ = 0%, GRADE: LOW), and 24 h postoperatively (MD: −0.58, 95% CI: −1.06 to −0.10, *I*^2^ = 72%, GRADE: VERY LOW). However, there was no significant difference between the two groups in the VAS scores (at rest) at 48 h postoperatively (MD: −0.48, 95% CI: −0.96 to 0.00, *I*^2^ = 77%, GRADE: VERY LOW) and 72 h postoperatively (MD: −0.18, 95% CI: −0.37 to 0.00, *I*^2^ = 0%, GRADE: MODERATE).

VAS scores (motion) were investigated in two RCTs involving 150 patients (10, 13). As shown in Fig. 3B, the meta-analysis demonstrated that the VAS scores (motion) were significantly lower in the MgSO_4_ group than the control group at 12 h postoperatively (MD: −0.63, 95% CI: −0.85 to −0.40, *I*^2^ = 0%, GRADE: LOW), 24 h postoperatively (MD: −0.53, 95% CI: −0.73 to −0.34, *I*^2^ = 0%, GRADE: LOW), and 48 h postoperatively (MD: −0.35, 95% CI: −0.54 to −0.16, *I*^2^ = 63%, GRADE: VERY LOW). However, there was no significant difference between the two groups in the VAS score (motion) at 48 h postoperatively (MD: −0.17, 95% CI: −0.35–0.01, *I*^2^ = 0%, GRADE: MODERATE).

### Morphine consumption and time to first rescue analgesia after total knee arthroplasty

**Figure 4:**
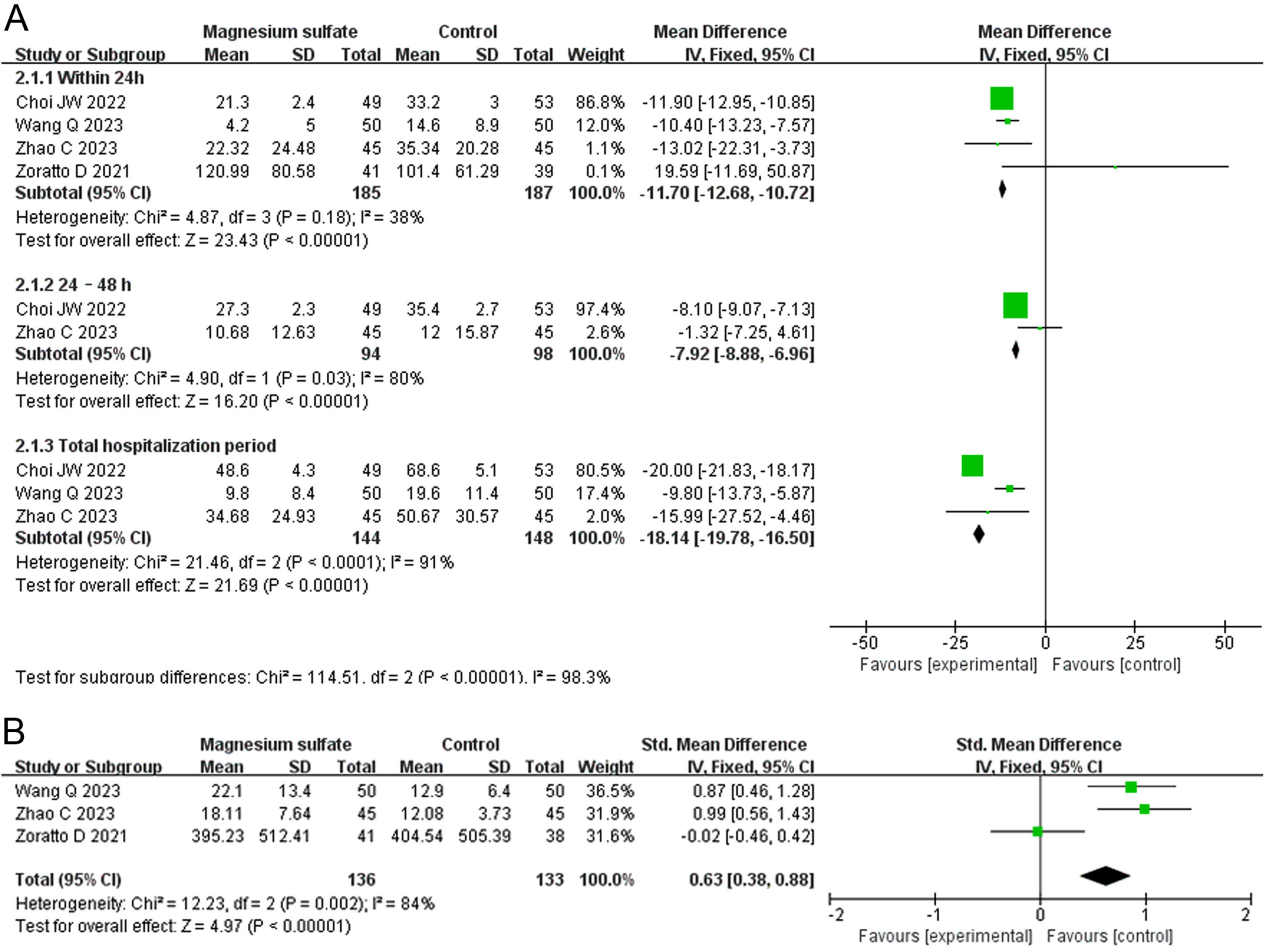
Forest plots displaying the mean differences between the magnesium sulfate and control groups in A) morphine consumption and B) time to first rescue analgesia after total knee arthroplasty. Green squares with horizontal lines represent the mean differences and 95% CIs for each trial. Black tiles serve as graphical representations of the mean differences at distinct time intervals or the indicators (12, 13, 14, 15).

Morphine consumption was investigated in four RCTs involving 372 patients (11, 12, 13, 14). As shown in Fig. 4A, the meta-analysis demonstrated that the morphine consumption was significantly lower in the MgSO_4_ group than the control group within 24 h postoperatively (MD: −11.70, 95% CI: −12.68 to −10.72 mg, *I*^2^ = 38%, GRADE: LOW), 24–48 h postoperatively (MD: −7.92, 95% CI: −8.88 to −6.96 mg, *I*^2^ = 80%, GRADE: VERY LOW), and during the total hospitalization period (MD −18.14, 95% CI: −19.78 to −16.50 mg, *I*^2^ = 91%, GRADE: VERY LOW).

The time to first rescue analgesia after TKA was reported in three RCTs involving 269 patients (12, 13, 14). As shown in Fig. 4B, the meta-analysis demonstrated that the time to first rescue analgesia after TKA was significantly shorter in the MgSO_4_ group than the control group (standardized MD: 0.63, 95% CI: 0.38–0.88, *I*^2^ = 84%, GRADE: VERY LOW).

### Knee joint function

**Figure 5:**
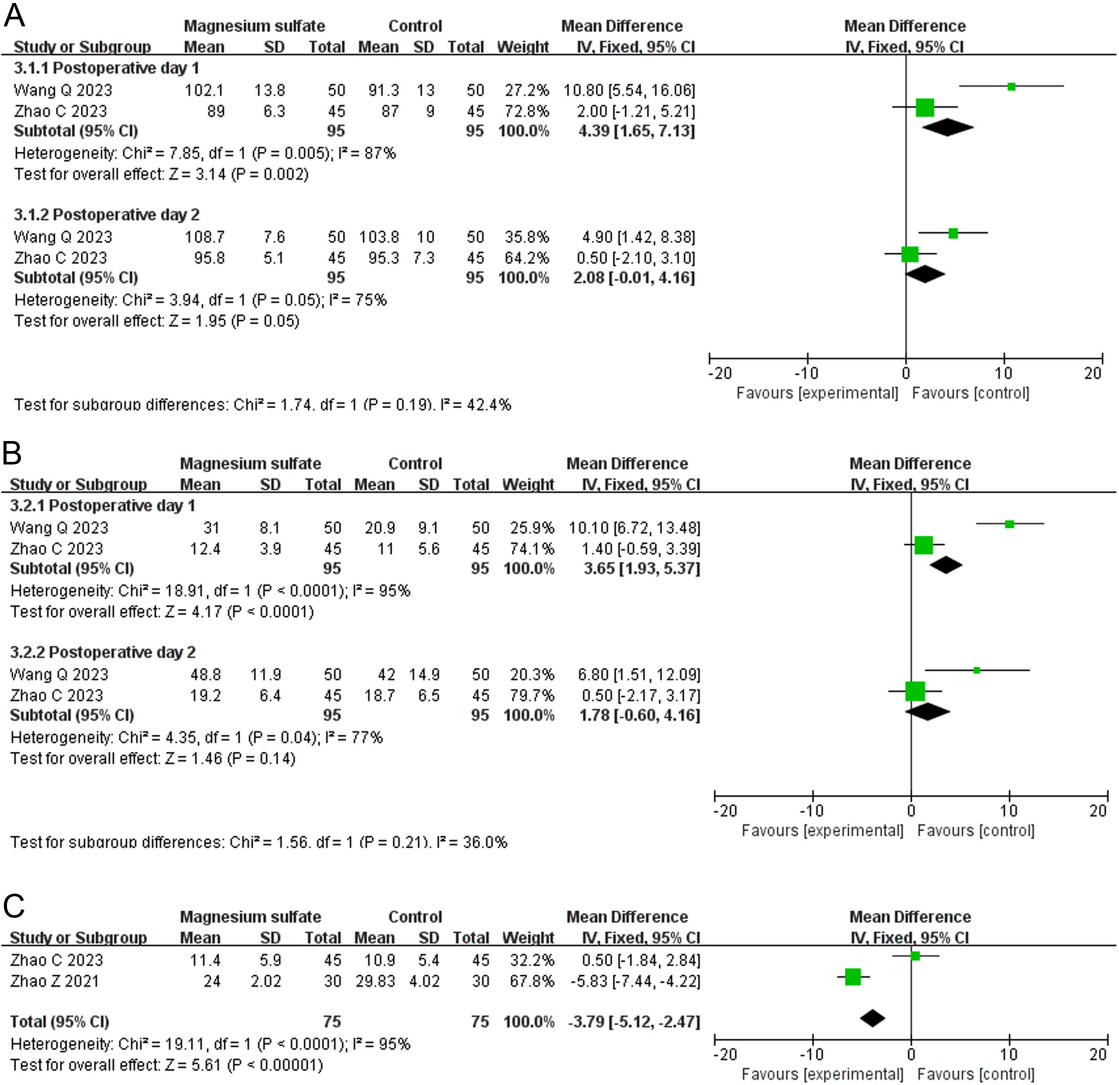
Forest plots displaying the mean differences between the magnesium sulfate and control groups on postoperative days 1 and 2 in the A) knee range of motion, B) daily mobilization distance, and C) time to first straight leg raising. Green squares with horizontal lines represent the mean differences and 95% CIs for each trial. Black tiles serve as graphical representations of the mean differences at distinct time intervals or the indicators (11, 13, 14).

The knee range of motion was reported in two RCTs involving 190 patients (12, 13). As shown in Fig. 5, the meta-analysis demonstrated that the knee range of motion on postoperative day 1 was significantly greater in the MgSO_4_ group than the control group (MD: 4.39, 95% CI: 1.65–7.13, *I*^2^ = 87%, GRADE: VERY LOW). However, there was no significant difference between the two groups in the knee range of motion on postoperative day 2 (MD: 2.08, 95% CI: −0.01–4.16, *I*^2^ = 75%, GRADE: VERY LOW).

The daily mobilization distance was reported in two RCTs involving 190 patients (10, 13). As shown in Fig. 5, the meta-analysis demonstrated that the MgSO_4_ group had a significantly greater daily mobilization distance on postoperative day 1 than the control group (MD: 3.65, 95% CI: 1.93–5.37, *I*^2^ = 95%, GRADE: VERY LOW). However, there was no significant difference between the two groups in the daily mobilization distance on postoperative day 2 (MD: 1.78, 95% CI: −0.60–4.16, *I*^2^ = 77%, GRADE: VERY LOW).

The time to first straight leg raising after TKA was reported in two RCTs involving 150 patients (10, 13). As shown in Fig. 5, the meta-analysis demonstrated that the MgSO_4_ group had a significantly shorter daily mobilization distance than the control group (MD −3.79, 95% CI: −5.12 to −2.47, *I*^2^ = 95%, GRADE: VERY LOW).

### Length of hospital stay

**Figure 6:**
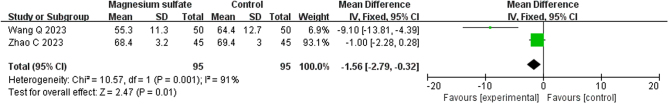
Forest plot displaying the mean difference between the magnesium sulfate and control groups in the length of hospital stay. Green squares with horizontal lines represent the mean differences and 95% CIs for each trial. Black tiles serve as graphical representations of the mean differences of the indicators (13, 14).

The postoperative LOS was reported in two RCTs involving 190 patients (12, 13). As shown in Fig. 6, the meta-analysis demonstrated that the MgSO_4_ group had a significantly shorter LOS than the control group (MD: −1.56, 95% CI: −2.79 to −0.32 h, *I*^2^ = 91%, GRADE: VERY LOW).

### Surgical complications

**Figure 7:**
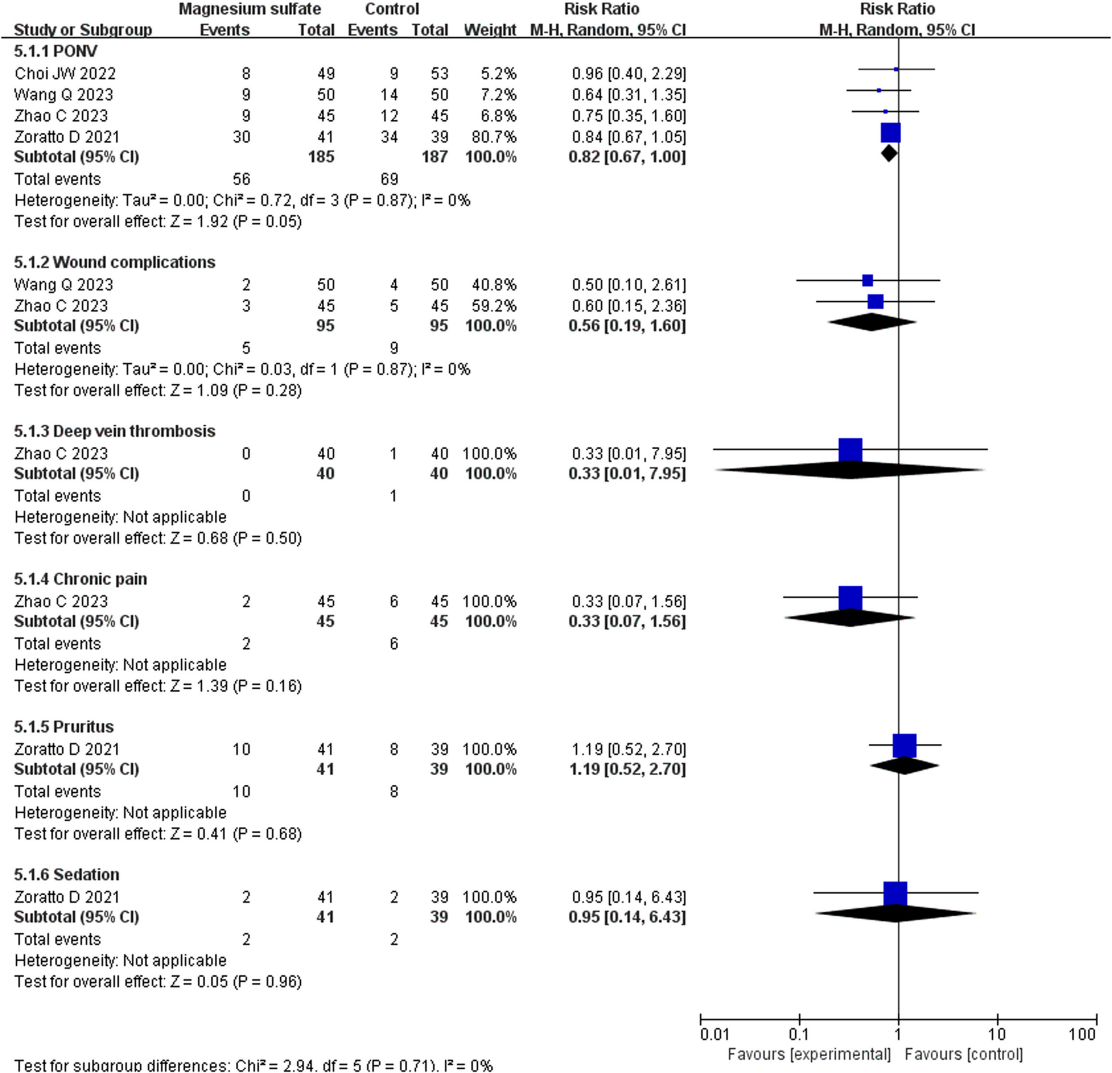
Forest plots displaying the mean differences in surgical complications between the magnesium sulfate and control groups. Green squares with horizontal lines represent the mean differences and 95% CIs for each trial. Black tiles serve as graphical representations of the mean differences of the indicators (12, 13, 14, 15).

The surgical complications are shown in Fig. 7.

PONV was reported in four RCTs involving 372 patients (11, 12, 13, 14). Meta-analysis demonstrated that there was no significant difference between the MgSO_4_ and control groups in the incidence of PONV (RR: 0.82, 95% CI: 0.67–1.00, *I*^2^ = 0%, GRADE: LOW).

Wound complications were reported in two RCTs involving 190 patients (12, 13). Meta-analysis demonstrated that there was no significant difference between the MgSO_4_ and control groups in the incidence of wound complications (RR: 0.56, 95% CI: 0.19–1.60, *I*^2^ = 0%, GRADE: VERY LOW).

Deep vein thrombosis was reported in one RCT involving 80 patients (13). Meta-analysis demonstrated that there was no significant difference between the MgSO_4_ and control groups in the incidence of deep vein thrombosis (RR: 0.33, 95% CI: 0.01–7.95, GRADE: VERY LOW).

Chronic pain was reported in one RCT involving 80 patients (13). Meta-analysis demonstrated that there was no significant difference between the MgSO_4_ and control groups in the incidence of chronic pain (RR: 0.33, 95% CI: 0.07–1.56, GRADE: VERY LOW).

Pruritus was reported in one RCT involving 80 patients (14). Meta-analysis demonstrated that there was no significant difference between the MgSO_4_ and control groups in the incidence of pruritus (RR: 1.19, 95% CI: 0.52–2.70, GRADE: LOW).

Sedation was reported in one RCT involving 80 patients (14). Meta-analysis demonstrated that there was no significant difference between the MgSO_4_ and control groups in the incidence of sedation (RR: 0.95, 95% CI: 0.14–6.43, GRADE: VERY LOW).

## Discussion

The use of multimodal pain management protocols after TKA has the ability to decrease pain scores, curtail the duration of hospitalization, accelerate recovery, and enhance patient satisfaction (21). Effective perioperative analgesia plays a crucial role in facilitating early return to exercise and rapid recovery after TKA (2). Magnesium causes significant analgesic effects that can decrease the required dose of opioids (22). Nowadays, MgSO_4_ is used as an effective analgesic adjuvant for various types of postoperative pain (23). In the pursuit of enhancing analgesia and prolonging the duration of peripheral analgesia, researchers began to investigate the addition of MgSO_4_ to the formulation of peripheral anesthetic cocktails. Recent studies have begun to discuss the addition of MgSO_4_ to the formulation of peripheral anesthetic cocktails in TKA, but the results are still controversial. Therefore, our meta-analysis and systematic review provide the first comprehensive overview of RCTs evaluating the addition of MgSO_4_ to the formulation of peripheral anesthetic cocktails used in TKA.

The effectiveness of analgesia for TKA may vary significantly depending on the composition of drugs in the peripheral analgesic cocktail. Presently, peripheral cocktail formulations predominantly comprise amide peripheral anesthetics in combination with glucocorticoids (24). However, commonly used amide peripheral anesthetics, such as ropivacaine and bupivacaine, typically do not offer prolonged analgesia, as their efficacy tends to diminish within approximately 10 h (25). MgSO_4_ is an NMDA receptor antagonist that can reduce the postoperative requirement for anesthetics and analgesics (26). It is suggested that the antinociceptive effect of magnesium can be harnessed to inhibit NMDA receptors (22). NMDA receptors play an important role in the transmission of central pain information and the regulation of acute hyperalgesia (27). Recent studies have shown that NMDA receptors also exist in peripheral tissues, such as muscles, skin, and joints, and play an important role in the transmission of nociceptive signals (28). Recent research has explored the incorporation of MgSO_4_ into peripheral anesthetics to enhance and potentially extend the duration of analgesia after various nerve blocks (9, 29). In addition, MgSO_4_ reportedly has the potential to augment and prolong the effects of ropivacaine-induced brachial plexus blocks (30). MgSO_4_ may exhibit favorable effects in TKA when used as an adjunct drug within a topical cocktail, but there is currently no evidence-based medicine to support this. Our meta-analysis revealed that the incorporation of MgSO_4_ into a peripheral analgesic cocktail is associated with a substantial extension of the analgesic duration, lasting for nearly 2 days after TKA. However, the analgesic effect gradually diminishes over time.

A primary objective in TKA is achieving optimal postoperative analgesia while simultaneously minimizing opioid consumption (31). Our meta-analysis suggests that the incorporation of MgSO_4_ into the peripheral analgesic cocktail has the potential to reduce opioid consumption. This is consistent with the finding that MgSO_4_ enhances analgesia. In fact, the improvement in pain management and reduction in opioid consumption after the addition of MgSO_4_ can be attributed to its analgesic properties (32). This finding is consistent with previous studies that have demonstrated the role of MgSO_4_ in improving pain outcomes after various surgical procedures (33).

The restoration of knee joint function after TKA has great clinical significance. Early functional exercise is essential to minimize joint stiffness, deep vein thrombosis, postoperative infections, and other complications due to inactivity (34). Functional recovery after TKA is influenced by various factors, including age, body mass index, operative factors, and pain. The knee function of the MgSO_4_ group exhibited a significant improvement compared with the control group within the first day after surgery, aligning with the duration of analgesia. These results suggest that the analgesic effect of MgSO_4_ may promote early postoperative ambulation and joint function recovery, contributing to enhanced patient mobility and rehabilitation.

Regarding adverse events, our analysis did not find any significant differences between the MgSO_4_ and control groups in terms of PONV, wound complications, deep vein thrombosis, chronic pain, pruritus, and sedation. Adverse effects of topical magnesium have been very rarely reported, with some studies describing an increased incidence of nausea (35). This is also one of the concerns with intravenous administration of MgSO_4_. However, our findings did not reveal any significant difference between the MgSO_4_ and control groups in the occurrence of PONV. There are two possible reasons for this phenomenon. First, the prominent cause of PONV is widely attributed to the administration of opioids. Thus, the potential of MgSO_4_ to decrease opioid usage may counterbalance the detrimental effects associated with magnesium supplementation. Secondly, adding MgSO_4_ to the peripheral analgesic cocktail, rather than intravenous MgSO_4_ administration, may decrease the incidence of adverse reactions. The present findings indicate that adding MgSO_4_ to the peripheral analgesic cocktail is well tolerated and safe for use in patients undergoing TKA, further supporting its potential as a valuable adjunct in pain management protocols.

LOS is a crucial metric for assessing enhanced recovery after TKA. Interestingly, our meta-analysis suggested that the addition of MgSO_4_ to the peripheral analgesic cocktail may reduce the LOS. This is possibly due to the analgesic effect of MgSO_4_ and the related decrease in opioid consumption, leading to faster recovery.

Our study shows that peripheral MgSO_4_ is effective in achieving analgesia after TKA. Researchers have suggested that the antinociceptive properties of magnesium are linked to its capacity to inhibit NMDA receptors. The function of NMDA receptors is correlated with magnesium levels. In a physiological context, magnesium blocks the ion channel on NMDA receptors, obstructing the entry of extracellular calcium ions into the cell and thereby averting secondary neuronal changes (36). This mechanism has the potential to impede central sensitization associated with nociception (37), which is found in patients with osteoarthritis (38). Therefore, MgSO_4_ may exert an analgesic effect by modulating central sensitization in patients who have undergone TKA, which may explain the results of our meta-analysis.

The present study represents the first systematic review and meta-analysis of the literature evaluating the impact of incorporating MgSO_4_ into a peripheral analgesic cocktail. The strengths of this meta-analysis include the extensive literature search, inclusion of updated literature, and comprehensive investigation of the effect of topical MgSO_4_ for analgesia in TKA. Moreover, we conducted meta-analyses and used the Recommendations Assessment, Development and Evaluation system to assess the evidence. However, our study also has several limitations. First, the numbers of included studies and patients were relatively small, which may have affected the overall statistical power and generalizability of our findings. Secondly, the unavoidable heterogeneity in outcomes requires subgroup analysis and meta-regression. However, owing to the limited number of included studies, the data available for analysis remained inadequate. Thus, a random-effects model was used for all outcomes to enhance the robustness of our findings. Thirdly, owing to the presently restricted data availability, our evaluation exclusively assessed the impact of MgSO_4_ as a supplementary component within a peripheral analgesic cocktail. However, our study did not evaluate the individual analgesic effects of topical MgSO_4_ as a standalone medication, nor does it establish the optimal dosage of MgSO_4_ when used as an adjunct. Future studies with larger sample sizes and standardized protocols are warranted to further validate our results.

Despite these limitations, the present systematic review and meta-analysis provides an up-to-date overview of the impact of topical MgSO_4_, a strategy that is not widely known, for analgesia in TKA. Our meta-analysis suggests that the addition of MgSO_4_ to the peripheral analgesic cocktail significantly enhances the analgesic efficacy in the early postoperative period (within 2 days) following TKA and decreases opioid consumption without increasing the incidence of adverse reactions. The use of MgSO_4_ as an adjunct to traditional analgesic strategies may offer a promising approach for optimizing pain management in TKA. These findings may suggest a new strategy for pain management in the early postoperative period of TKA. However, given the low or very low quality of the studies analyzed for outcomes, there is a need for more high-quality RCTs exploring the long-term addition of MgSO_4_ to the peripheral analgesic cocktail in order to enhance analgesic efficacy after TKA. Additionally, the dosage of MgSO_4_ used in the included literature was inconsistent, necessitating further studies to determine the optimal dosage. Given that studies have documented sex-based differences in pain perception (39), further research is also required to address potential confounding factors.

## Conclusion

The findings of this meta-analysis suggest that MgSO_4_ considerably augments the analgesic potency of a peripheral analgesic cocktail during the initial postoperative period (within 2 days) following TKA. Furthermore, the addition of MgSO_4_ appears to decrease opioid consumption without increasing the incidence of adverse reactions.

## Supplementary Materials

Supplementary Tables

## ICMJE Conflict of Interest Statement

The authors declare that there is no conflict of interest that could be perceived as prejudicing the impartiality of the study reported.

## Funding Statement

This paper was supported by Traditional Chinese Medicine Inheritance and Innovative Talent Project (Zhongjing Project) Top-notch Chinese Medicine Talents Training Project (Yuwei Chinese Medicine Letter (2021) no. 15).

## References

[1] Shichman I Roof M Askew N Nherera L Rozell JC Seyler TM & Schwarzkopf R. Projections and epidemiology of primary hip and knee arthroplasty in Medicare patients to 2040–2060. JBJS Open Access 2023 8 e22.00112. (10.2106/JBJS.OA.22.00112) PMC9974080 36864906

[2] Karam JA Schwenk ES & Parvizi J. An update on multimodal pain management after total joint arthroplasty. Journal of Bone and Joint Surgery 2021 103 1652–1662. (10.2106/JBJS.19.01423) 34232932

[3] Yu S Dundon J Solovyova O Bosco J & Iorio R. Can multimodal pain management in TKA eliminate patient-controlled analgesia and femoral nerve blocks? Clinical Orthopaedics and Related Research 2018 476 101–109. (10.1007/s11999.0000000000000018) 29529623 PMC5919240

[4] Li Z Li Z Cheng K & Weng X. The efficacy and safety of glucocorticoid on periarticular infiltration analgesia in total knee arthroplasty: a systematic review and meta-analysis of randomized controlled trials. Journal of Arthroplasty 2021 36 3340–3350. (10.1016/j.arth.2021.03.056) 33926778

[5] Pepper AM Mercuri JJ Behery OA & Vigdorchik JM. Total hip and knee arthroplasty perioperative pain management: what should be in the cocktail. JBJS Reviews 2018 6 e5. (10.2106/JBJS.RVW.18.00023) 30562208

[6] Wang Q Tan G Mohammed A Zhang Y Li D Chen L & Kang P. Adding corticosteroids to periarticular infiltration analgesia improves the short-term analgesic effects after total knee arthroplasty: a prospective, double-blind, randomized controlled trial. Knee Surgery, Sports Traumatology, Arthroscopy 2021 29 867–875. (10.1007/s00167-020-06039-9) 32361928

[7] Wang Q Sun J Hu Y Zeng Y Hu J Yang J & Kang P. Effects of morphine on periarticular infiltration analgesia in total knee arthroplasty: a prospective, double-blind, randomized controlled trial. International Orthopaedics 2020 44 2587–2595. (10.1007/s00264-020-04700-z) 32705319

[8] Jæger P Zaric D Fomsgaard JS Hilsted KL Bjerregaard J Gyrn J Mathiesen O Larsen TK & Dahl JB. Adductor canal block versus femoral nerve block for analgesia after total knee arthroplasty: a randomized, double-blind study. Regional Anesthesia and Pain Medicine 2013 38 526–532. (10.1097/AAP.0000000000000015) 24121608

[9] Li M Jin S Zhao X Xu Z Ni X Zhang L & Liu Z. Does magnesium sulfate as an adjuvant of local anesthetics facilitate better effect of perineural nerve blocks? A meta-analysis of randomized controlled trials. Clinical Journal of Pain 2016 32 1053–1061. (10.1097/AJP.0000000000000356) 26889623

[10] Zhao Z Zhang X Peng H Li W Liu H & Wu H. Magnesium sulfate combined with a levobupivacaine periarticular cocktail for analgesia in the early postoperative period after total knee arthroplasty. Journal of Knee Surgery 2021 34 1463–1468. (10.1055/s-0040-1710364) 32434237

[11] Choi JW Lahori A Merlo JA Gill O Ghoddoussi F Patel KM Desai RG Hakim J Zatkoff J & Krishnan S. Adductor canal blocks with bupivacaine and magnesium after same-day discharge total knee arthroplasty improve postoperative pain relief and decrease opioid consumption: a prospective randomized controlled trial. Clinical Journal of Pain 2022 38 388–395. (10.1097/AJP.0000000000001036) 35440521

[12] Wang Q Zhao C Hu J Ma T Yang J & Kang P. Efficacy of a modified cocktail for periarticular local infiltration analgesia in total knee arthroplasty: a prospective, double-blinded, randomized controlled trial. Journal of Bone and Joint Surgery 2023 105 354–362. (10.2106/JBJS.22.00614) 36856693

[13] Zhao C Wang L Chen L Wang Q & Kang P. Effects of magnesium sulfate on periarticular infiltration analgesia in total knee arthroplasty: a prospective, double-blind, randomized controlled trial. Journal of Orthopaedic Surgery and Research 2023 18 301. (10.1186/s13018-023-03790-w) 37060089 PMC10105472

[14] Zoratto D Phelan R Hopman WM Wood GCA Shyam V DuMerton D Shelley J McQuaide S Kanee L Ho AMH, *et al.* Adductor canal block with or without added magnesium sulfate following total knee arthroplasty: a multi-arm randomized controlled trial. Canadian Journal of Anesthesia 2021 68 1028–1037. (10.1007/s12630-021-01985-5) 34041719

[15] Higgins JPT Thomas J Chandler J Cumpston M Li T Page MJ & Welch VA (editors). Cochrane Handbook for Systematic Reviews of Interventions version 6.4 (updated August 2023). Cochrane, 2023. Available from www.training.cochrane.org/handbook

[16] Moher D Liberati A Tetzlaff J Altman DG & PRISMA Group. Preferred Reporting Items for Systematic Reviews and Meta-Analyses: the PRISMA statement. Journal of Clinical Epidemiology 2009 62 1006–1012. (10.1016/j.jclinepi.2009.06.005) 19631508

[17] Nielsen S Degenhardt L Hoban B & Gisev N. A synthesis of oral morphine equivalents (OME) for opioid utilisation studies. Pharmacoepidemiology and Drug Safety 2016 25 733–737. (10.1002/pds.3945) 26693665

[18] Phi L Ajaj R Ramchandani MH Brant XM Oluwadara O Polinovsky O Moradi D Barkhordarian A Sriphanlop P Ong M, *et al.* Expanding the Grading of Recommendations Assessment, Development, and Evaluation (Ex-GRADE) for evidence-based clinical recommendations: validation study. Open Dentistry Journal 2012 6 31–40. (10.2174/1874210601206010031) 22303416 PMC3269009

[19] Shin HJ Kim EY Na HS Kim TK Kim MH & Do SH. Magnesium sulphate attenuates acute postoperative pain and increased pain intensity after surgical injury in staged bilateral total knee arthroplasty: a randomized, double-blinded, placebo-controlled trial. British Journal of Anaesthesia 2016 117 497–503. (10.1093/bja/aew227) 28077538

[20] Chen Y Zhang Y Zhu YL & Fu PL. Efficacy and safety of an intra-operative intra-articular magnesium/ropivacaine injection for pain control following total knee arthroplasty. Journal of International Medical Research 2012 40 2032–2040. (10.1177/030006051204000548) 23206490

[21] Summers S Mohile N McNamara C Osman B Gebhard R & Hernandez VH. Analgesia in total knee arthroplasty: current pain control modalities and outcomes. Journal of Bone and Joint Surgery 2020 102 719–727. (10.2106/JBJS.19.01035) 31985507

[22] Soleimanpour H Imani F Dolati S Soleimanpour M & Shahsavarinia K. Management of pain using magnesium sulphate: a narrative review. Postgraduate Medicine 2022 134 260–266. (10.1080/00325481.2022.2035092) 35086408

[23] Paula-Garcia WN Oliveira-Paula GH de Boer HD & Garcia LV. Lidocaine combined with magnesium sulfate preserved hemodynamic stability during general anesthesia without prolonging neuromuscular blockade: a randomized, double-blind, controlled trial. BMC Anesthesiology 2021 21 91. (10.1186/s12871-021-01311-y) 33773580 PMC8004390

[24] Jackson JD Cotton L Turkington M Leblanc D & Kelley S. Physical and chemical compatibility of extended-release triamcinolone acetonide (TA-ER) with common local anesthetics. Advances in Therapy 2019 36 652–661. (10.1007/s12325-019-0878-2) 30706409 PMC6824335

[25] Xiao J Cai MH Wang XR He P & Wang XR. Time course of action and pharmacokinetics of ropivacaine in adult and elderly patients following combined lumbar plexus-sciatic nerve block. International Journal of Clinical Pharmacology and Therapeutics 2010 48 608–613. (10.5414/cpp48608) 20860914

[26] Faiz SHR Rahimzadeh P Sakhaei M Imani F & Derakhshan P. Anesthetic effects of adding intrathecal neostigmine or magnesium sulphate to bupivacaine in patients under lower extremities surgeries. Journal of Research in Medical Sciences 2012 17 918–922. (10.4103/1119-3077.104540) 23825989 PMC3698648

[27] Kreutzwiser D & Tawfic QA. Expanding role of NMDA receptor antagonists in the management of pain. CNS Drugs 2019 33 347–374. (10.1007/s40263-019-00618-2) 30826987

[28] Elsharkawy RA Farahat TE & Abdelhafez MS. Analgesic effect of adding magnesium sulfate to epidural levobupivacaine in patients with pre-eclampsia undergoing elective cesarean section. Journal of Anaesthesiology, Clinical Pharmacology 2018 34 328–334. (10.4103/joacp.JOACP_1_18) 30386015 PMC6194846

[29] Lee AR Yi HW Chung IS Ko JS Ahn HJ Gwak MS Choi DH & Choi SJ. Magnesium added to bupivacaine prolongs the duration of analgesia after interscalene nerve block. Canadian Journal of Anesthesia 2012 59 21–27. (10.1007/s12630-011-9604-5) 22012543

[30] Deshpande JP & Patil KN. Evaluation of magnesium as an adjuvant to ropivacaine-induced axillary brachial plexus block: a prospective, randomised, double-blind study. Indian Journal of Anaesthesia 2020 64 310–315. (10.4103/ija.IJA_833_19) 32489206 PMC7259414

[31] Tong QJ Lim YC & Tham HM. Comparing adductor canal block with local infiltration analgesia in total knee arthroplasty: a prospective, blinded and randomized clinical trial. Journal of Clinical Anesthesia 2018 46 39–43. (10.1016/j.jclinane.2018.01.014) 29414612

[32] Malik KM Imani F Beckerly R & Chovatiya R. Risk of opioid use disorder from exposure to opioids in the perioperative period: a systematic review. Anesthesiology and Pain Medicine 2020 10 e101339. (10.5812/aapm.101339) 32337175 PMC7158240

[33] Peng YN Sung FC Huang ML Lin CL & Kao CH. The use of intravenous magnesium sulfate on postoperative analgesia in orthopedic surgery: a systematic review of randomized controlled trials. Medicine (Baltimore) 2018 97 e13583. (10.1097/MD.0000000000013583) 30558026 PMC6319973

[34] Jakobsen TL Kehlet H Husted H Petersen J & Bandholm T. Early progressive strength training to enhance recovery after fast-track total knee arthroplasty: a randomized controlled trial. Arthritis Care and Research 2014 66 1856–1866. (10.1002/acr.22405) 25074397

[35] Akhondzade R Nesioonpour S Gousheh M Soltani F & Davarimoghadam M. The effect of magnesium sulfate on postoperative pain in upper limb surgeries by supraclavicular block under ultrasound guidance. Anesthesiology and Pain Medicine 2017 7 e14232. (10.5812/aapm.14232) 28924560 PMC5594567

[36] Zhong HY & Zhang WP. Effect of intravenous magnesium sulfate on bupivacaine spinal anesthesia in preeclamptic patients. Biomedicine and Pharmacotherapy 2018 108 1289–1293. (10.1016/j.biopha.2018.09.157) 30372830

[37] Park R Ho AMH Pickering G Arendt-Nielsen L Mohiuddin M & Gilron I. Efficacy and safety of magnesium for the management of chronic pain in adults: a systematic review. Anesthesia and Analgesia 2020 131 764–775. (10.1213/ANE.0000000000004673) 32049671

[38] Arendt-Nielsen L Nie H Laursen MB Laursen BS Madeleine P Simonsen OH & Graven-Nielsen T. Sensitization in patients with painful knee osteoarthritis. Pain 2010 149 573–581. (10.1016/j.pain.2010.04.003) 20418016

[39] Bartley EJ & Fillingim RB. Sex differences in pain: a brief review of clinical and experimental findings. British Journal of Anaesthesia 2013 111 52–58. (10.1093/bja/aet127) 23794645 PMC3690315

