# Supplementary material for: Magnesium sulfate enhances the effect of the peripheral analgesic cocktail in total knee arthroplasty: a systematic review and meta-analysis of randomized controlled trials
Source: EFORT Open Rev. 2024 Sep 2;9(9):896–907. doi: 10.1530/EOR-23-0185 (PMC11457811; doi:10.1530/EOR-23-0185)
Supplement: Supplementary Tables [file EOR-23-0185supplementary_tables.pdf]

Supplemental Table

Supplemental Table 1: Search strategy applied in the PubMed database

Table 1 Search strategy as applied in PubMed

#1	Magnesium Sulfate
#2	Magnesium
#3	Magnesium Sulfate, Heptahydrate
#4	1 or 2 or 3
#5	Arthroplasty, Replacement, Knee
#6	Arthroplasty, Knee Replacement
#7	Knee Replacement Arthroplasty
#8	Knee Arthroplasty, Total
#9	Arthroplasty, Total Knee
#10	Total Knee Arthroplasty
#11	Replacement, Total Knee
#12	Total Knee Replacement
#13	5 or 6 or 7 or 8 or 9 or 10 or 11 or 12
#14	4 and 13

Supplemental Table 2: Summary of the Grading of Recommendations Assessment, Development, and Evaluation (GRADE)

Outcomes

Author(s):
Date: 2023-09-11
Question: Should VAS(at rest) be used for total knee arthroplasty?
Settings:
Bibliography: . magnesium sulfate for total knee arthroplasty. Cochrane Database of Systematic Reviews [Year], Issue [Issue].

Quality assessment							No of patients		Effect		Quality	Importance
No of studies	Design	Risk of bias	Inconsistency	Indirectness	Imprecision	Other considerations	VAS(at rest)	Control	Relative (95% CI)	Absolute		
**VAS(at rest) - Postoperative 6h (Better indicated by lower values)**												
2	randomised trials	serious[1]	no serious inconsistency	no serious indirectness	no serious imprecision	none	75	75	-	MD 0.52 lower (0.77 to 0.26 lower)	⊕⊕⊕O MODERATE	CRITICAL
**VAS(at rest) - Postoperative 12h (Better indicated by lower values)**												
3	randomised trials	serious[1]	no serious inconsistency	no serious indirectness	serious[2]	none	108	105	-	MD 0.53 lower (0.74 to 0.31 lower)	⊕⊕OO LOW	CRITICAL
**VAS(at rest) - Postoperative 24h (Better indicated by lower values)**												
4	randomised trials	serious[3]	serious[4]	no serious indirectness	serious[2]	none	157	163	-	MD 0.58 lower (1.06 to 0.1 lower)	⊕OOO VERY LOW	CRITICAL
**VAS(at rest) - Postoperative 48h (Better indicated by lower values)**												
4	randomised trials	serious[3]	very serious[5]	no serious indirectness	very serious[2,6]	none	156	160	-	MD 0.48 lower (0.96 lower to 0 higher)	⊕OOO VERY LOW	CRITICAL
**VAS(at rest) - Postoperative 72h (Better indicated by lower values)**												
2	randomised trials	serious[1]	no serious inconsistency	no serious indirectness	no serious imprecision	none	75	75	-	MD 0.18 lower (0.37 lower to 0 higher)	⊕⊕⊕O MODERATE	CRITICAL
**VAS(motion) - Postoperative 12h (Better indicated by lower values)**												
2	randomised trials	serious[1]	no serious inconsistency	no serious indirectness	serious[2]	none	75	75	-	MD 0.63 lower (0.85 to 0.4 lower)	⊕⊕OO LOW	CRITICAL
**VAS(motion) - Postoperative 24h (Better indicated by lower values)**												
2	randomised trials	serious[1]	no serious inconsistency	no serious indirectness	serious[2]	none	75	75	-	MD 0.53 lower (0.73 to 0.34 lower)	⊕⊕OO LOW	CRITICAL
**VAS(motion) - Postoperative 48h (Better indicated by lower values)**												
2	randomised trials	serious[1]	serious[4]	no serious indirectness	serious[2]	none	75	75	-	MD 0.35 lower (0.54 to 0.16 lower)	⊕OOO VERY LOW	CRITICAL
**VAS(motion) - Postoperative 72h (Better indicated by lower values)**												
2	randomised trials	serious[3]	no serious inconsistency	no serious indirectness	no serious imprecision	none	75	75	-	MD 0.17 lower (0.35 lower to 0.01 higher)	⊕⊕⊕O MODERATE	CRITICAL

[1] lack of allocation concealment
[2] mean difference greater than 25%
[3] lack of blinding
[4] 50 < I2 < 75%
[5] I2≥75%
[6] 95% confidence interval cross the line

Author(s):
Date: 2023-09-11
Question: Morphine consumption for total knee arthroplasty
Settings:
Bibliography: . magnesium sulfate for total knee arthroplasty. Cochrane Database of Systematic Reviews [Year], Issue [Issue].

Quality assessment							No of patients		Effect		Quality	Importance
No of studies	Design	Risk of bias	Inconsistency	Indirectness	Imprecision	Other considerations	Morphine consumption	Control	Relative (95% CI)	Absolute		
**Morphine consumption - Within 24h (Better indicated by lower values)**												
4	randomised trials	serious[1]	no serious inconsistency	no serious indirectness	serious[2]	none	185	187	-	MD 11.7 lower (12.68 to 10.72 lower)	⊕⊕OO LOW	CRITICAL
**Morphine consumption - 24–48 h (Better indicated by lower values)**												
2	randomised trials	serious[3]	very serious[4]	no serious indirectness	no serious imprecision	none	94	98	-	MD 7.92 lower (8.88 to 6.96 lower)	⊕OOO VERY LOW	CRITICAL
**Morphine consumption - Total hospitalization period (Better indicated by lower values)**												
3	randomised trials	serious[3]	very serious[4]	no serious indirectness	serious[2]	none	144	148	-	MD 18.14 lower (19.78 to 16.5 lower)	⊕OOO VERY LOW	CRITICAL
**Time to frst rescue analgesia (Better indicated by lower values)**												
3	randomised trials	serious[3]	very serious[4]	no serious indirectness	serious[2]	none	136	133	-	SMD 0.63 higher (0.38 to 0.88 higher)	⊕OOO VERY LOW	CRITICAL

[1] lack of blinding
[2] mean difference greater than 25%
[3] lack of allocation concealment
[4] I2≥75%

Author(s):
Date: 2023-09-11
Question: Knee function for total knee arthroplasty
Settings:
Bibliography: . magnesium sulfate for total knee arthroplasty. Cochrane Database of Systematic Reviews [Year], Issue [Issue].

Quality assessment							No of patients		Effect		Quality	Importance
No of studies	Design	Risk of bias	Inconsistency	Indirectness	Imprecision	Other considerations	Knee function	Control	Relative (95% CI)	Absolute		
**knee range of motion - Postoperative day 1 (Better indicated by lower values)**												
2	randomised trials	serious[1]	very serious[2]	no serious indirectness	serious[3]	none	95	95	-	MD 4.39 higher (1.65 to 7.13 higher)	⊕OOO VERY LOW	IMPORTANT
**knee range of motion - Postoperative day 2 (Better indicated by lower values)**												
2	randomised trials	serious[1]	very serious[2]	no serious indirectness	serious[3]	none	95	95	-	MD 2.08 higher (0.01 lower to 4.16 higher)	⊕OOO VERY LOW	IMPORTANT
**Daily mobilization distance - Postoperative day 1 (Better indicated by lower values)**												
2	randomised trials	serious[1]	very serious[2]	no serious indirectness	serious[3]	none	95	95	-	MD 3.65 higher (1.93 to 5.37 higher)	⊕OOO VERY LOW	IMPORTANT
**Daily mobilization distance - Postoperative day 2 (Better indicated by lower values)**												
2	randomised trials	serious[1]	very serious[2]	no serious indirectness	very serious[3,4]	none	95	95	-	MD 1.78 higher (0.6 lower to 4.16 higher)	⊕OOO VERY LOW	IMPORTANT
**Time to frst straight-leg raising (Better indicated by lower values)**												
2	randomised trials	serious[5]	very serious[2]	no serious indirectness	serious[3]	none	75	75	-	MD 3.79 lower (5.12 to 2.47 lower)	⊕OOO VERY LOW	IMPORTANT

[1] lack of allocation concealment
[2] I2≥75%
[3] mean difference greater than 25%
[4] 95% confidence interval cross the line
[5] lack of blinding

Author(s):
Date: 2023-09-11
Question: Postoperative length of stay for total knee arthroplasty
Settings:
Bibliography: . magnesium sulfate for total knee arthroplasty. Cochrane Database of Systematic Reviews [Year], Issue [Issue].

Quality assessment							No of patients		Effect		Quality	Importance
No of studies	Design	Risk of bias	Inconsistency	Indirectness	Imprecision	Other considerations	Postoperative length of stay	Control	Relative (95% CI)	Absolute		
**Postoperative length of stay (Better indicated by lower values)**												
2	randomised trials	serious[1]	very serious[2]	no serious indirectness	serious[3]	none	95	95	-	MD 1.56 lower (2.79 to 0.32 lower)	⊕OOO VERY LOW	IMPORTANT

[1] lack of allocation concealment
[2] I2≥75%
[3] mean difference greater than 25%

Postoperative complications for total knee arthroplasty

Patient or population: patients with total knee arthroplasty
Settings:
Intervention: Postoperative complications

Outcomes	Illustrative comparative risks* (95% CI)		Relative effect (95% CI)	No of Participants (studies)	Quality of the evidence (GRADE)	Comments
	Assumed risk	Corresponding risk				
	Control	Postoperative complications				
complications - PONV	Study population		RR 0.82 (0.67 to 1)	372 (4 studies)	⊕⊕⊖⊖ low[1,2]	
	369 per 1000	303 per 1000 (247 to 369)				
	Moderate					
	273 per 1000	224 per 1000 (183 to 273)				
complications - Wound complications	Study population		RR 0.56 (0.19 to 1.6)	190 (2 studies)	⊕⊖⊖⊖ very low[1,2,3]	
	95 per 1000	53 per 1000 (18 to 152)				
	Moderate					
	96 per 1000	54 per 1000 (18 to 154)				
complications - Deep vein thrombosis	Study population		RR 0.33 (0.01 to 7.95)	80 (1 study)	⊕⊖⊖⊖ very low[1,2,3]	
	25 per 1000	8 per 1000 (0 to 199)				
	Moderate					
	25 per 1000	8 per 1000 (0 to 199)				
complications - Chronic pain	Study population		RR 0.33 (0.07 to 1.56)	90 (1 study)	⊕⊖⊖⊖ very low[1,2,3]	
	133 per 1000	44 per 1000 (9 to 208)				
	Moderate					
	133 per 1000	44 per 1000 (9 to 207)				
complications - Pruritus	Study population		RR 1.19 (0.52 to 2.7)	80 (1 study)	⊕⊕⊖⊖ low[2,4]	
	205 per 1000	244 per 1000 (107 to 554)				
	Moderate					
	205 per 1000	244 per 1000 (107 to 554)				
complications - Sedation	Study population		RR 0.95 (0.14 to 6.43)	80 (1 study)	⊕⊖⊖⊖ very low[2,3,4]	
	51 per 1000	49 per 1000 (7 to 330)				
	Moderate					
	51 per 1000	48 per 1000 (7 to 328)				

*The basis for the **assumed risk** (e.g. the median control group risk across studies) is provided in footnotes. The **corresponding risk** (and its 95% confidence interval) is based on the assumed risk in the comparison group and the **relative effect** of the intervention (and its 95% CI).

CI: Confidence interval; **RR:** Risk ratio;

GRADE Working Group grades of evidence
High quality: Further research is very unlikely to change our confidence in the estimate of effect.
Moderate quality: Further research is likely to have an important impact on our confidence in the estimate of effect and may change the estimate.
Low quality: Further research is very likely to have an important impact on our confidence in the estimate of effect and is likely to change the estimate.
Very low quality: We are very uncertain about the estimate.

[1] lack of allocation concealment

[2] 95% confidence interval cross the line

[3] risk ratio greater than 25%

[4] selective outcome reporting
